# Emergence of Bartonella quintana infection among homeless persons.

**DOI:** 10.3201/eid0202.960212

**Published:** 1996

**Authors:** L A Jackson, D H Spach

**Affiliations:** School of Public Health and Community Medicine, University of Washington, Seattle, Washington, USA.


### Emergence of *Bartonella quintana* Infection among Homeless Persons

*Bartonella quintana* has episodically emerged as a cause of infection among distinct and diverse populations during the 20th century. The organism was first identified as an important human pathogen during World War I when it caused epidemics of louse-borne trench fever that affected an estimated 1 million troops in Europe (1, 2). Trench fever was characterized by fever, rash, bone pain, and splenomegaly and ranged in severity from a mild flulike illness to a more severe, relapsing disease. *B. quintana* infections were rarely recognized from the end of World War II until the 1980s when the organism reemerged as an opportunistic pathogen among HIV-infected persons. In this population, *B. quintana* has been identified as a cause of bacillary angiomatosis, endocarditis, and bacteremia (3-5) and has been isolated from AIDS patients in France (6) and the United States (3-5).

In the 1990s, *B. quintana* has emerged among homeless persons in North America and Europe. In 1993, the organism was isolated from the blood specimens of 10 patients at a single hospital in Seattle, Washington, within a 6-month period (7). These patients had illnesses characterized by fever and persistent bacteremia. Endocarditis developed in two patients, one of whom required a heart valve replacement. All 10 patients had chronic alcoholism, eight were homeless, and the six who were tested for HIV infection were HIV-negative. These six were the first cases of invasive *B. quintana* infection among HIV-negative persons reported in the United States. Results of a case-control study indicated that the patients with *Bartonella* bacteremia were more likely than controls (other hospitalized patients from whom blood specimens were obtained at approximately the same time) to be homeless ($p = 0.001$), to have a history of alcohol abuse ($p = 0.001$), and to be nonwhite ($p = 0.007$). The isolates from the 10 patients were identical by polymerase chain reaction restriction-fragment-length polymorphism testing, which further suggests that the cases were epidemiologically linked. Patients' characteristics were obtained by retrospective medical record review, and at the time they sought treatment, three patients reported a recent cat scratch, five had scabies, and one had lice. More complete information, however, on patients' past exposures to animals and ectoparasites was not available.

In 1995, Drancourt and co-workers reported three cases of *B. quintana* endocarditis among HIV-negative, homeless, alcoholic men in France (8). One of the patients had reported contact with a dog, and one had reported contact with dogs and cats; however, a current or past history of infection with lice or scabies was not documented for any of the patients. In 1995, Stein and Raoult also reported serologic evidence of *B. quintana* infection in an HIV-negative, homeless man from Marseilles, who had a relapsing febrile illness and a history of louse infestation (9).

As a follow-up to the 1993 *B. quintana* outbreak in Seattle in 1994, we conducted a seroprevalence study of anti-*Bartonella* antibodies among patients at a community clinic in the "skid row" section of Seattle, which serves a primarily homeless and indigent population (10). The median age of the 192 patients included in the study was 45 years, 156 (81%) of the 192 were male, and 126 (66%) were classified as homeless. *B. quintana* IgG titers ≥ 64 were detected by an indirect fluorescence antibody assay (11) in 39 (20%) of the 192 clinic patients. In contrast, only 4 (2%) of 199 banked blood specimens from an age-matched and sex-matched comparison group of Seattle volunteer blood donors had titers ≥ 64 ($p < 0.001$). Among clinic patients, seropositivity (titer ≥ 64) was associated by univariate analysis with older age, homelessness (relative risk [RR] 2.0; 95% confidence interval [CI] 1.0-4.1), alcohol abuse (RR, 2.5; 95% CI 1.4-4.2), smoking (RR, 2.0; 95% CI 1.2 -3.4), and injection drug use (RR, 2.5; 95% CI 1.3-4.8). By multivariate analysis, only alcohol abuse remained independently associated with seropositivity (odds ratio 3.3; 95% CI 1.6-6.9), and of 39 seropositive patients, 21 (54%) had a history of chronic alcoholism. Reliable data on past exposure to animals or ectoparasites were also not available for patients in this study.

The study was limited by the well-described cross-reactivity of the assay between *Bartonella* species (12, 13), and most (62%) clinic patients with *B. quintana* titers ≥ 64 also had titers ≥ 64 to *B. henselae*. It is, therefore, possible that some of the seropositive patients may have been exposed to *Bartonella* species other than *B. quintana*. These findings do, however, show that a surprisingly high proportion of clinic patients without a history of documented *Bartonella* infection had detectable anti-*Bartonella* antibodies and may have been exposed to *B. quintana*.

Multiple factors, including those related to disease transmission, host susceptibility, and ability to detect the organism, have likely contributed to the emergence of *B. quintana* infection among the homeless. Transmission of *B. quintana* from human to human by the body louse, *Pediculus humanus*, has been experimentally documented (1) and is believed to have been the predominant mode of transmission of epidemic trench fever in World Wars I and II. Lice reside primarily in the seams of clothing and are easily killed by immersion in water 50°C or warmer (14), which explains the propensity for louse-borne infections among displaced persons or wartime troops. Although these reports of *B. quintana* infection among homeless persons lack sufficient information to conclusively determine the disease vector, louse-borne infection remains a plausible hypothesis. Lice, however, have not been associated with bacillary angiomatosis among AIDS patients, although exposure to cats (and, therefore, possibly to fleas) has been associated with bacillary angiomatosis and bacillary peliosis caused by *Bartonella* species (15) and with cat-scratch disease caused by *B. henselae* (16). Thus, it is possible that *B. quintana* infection is spread among homeless persons by as yet unidentified vectors or reservoirs.

Homeless persons are also at risk for non-vectorborne infectious diseases. An increased risk for tuberculosis in this population is well documented (17, 18), and outbreaks of meningococcal disease (19, 20), pneumococcal disease (21), and diphtheria (22, 23) have been reported. It is likely that factors such as crowding, altered immunity due to alcoholism or other co-existing health problems, and inadequate or infrequent access to medical care affect the transmission and spread of infectious diseases among the homeless. Previous studies have shown that the clinical response to a standard inoculum of *B. quintana* varies substantially in experimental study patients (1); this variation indicates that host factors are likely important determinants of the risk for clinical infection following exposure to the organism.

Although cases of *B. quintana* bacteremia among homeless persons have thus far been reported only from France and Seattle, Washington, the problem is probably not confined to these locations. *B. quintana* is a fastidious and slow-growing bacterium that generally requires special culturing techniques for isolation (3-5, 24), and many clinical laboratories do not routinely use blood culturing methods that are sensitive for isolating this organism. Moreover, *B. quintana* infection can result in a broad range of often nonspecific clinical manifestations (1, 3-5); therefore, case-patients evaluated for suspected bacteremia may represent only a small proportion of infected persons, as suggested by the results of the Seattle seroprevalence survey. To better define the geographic distribution and prevalence of *B. quintana* infection among homeless populations, a heightened awareness for this infection on the part of clinicians and the use of appropriate culture techniques by microbiology laboratories serving this population are needed. In addition, more specific serologic tests would aid in the diagnosis and assessment of the epidemiologic characteristics of *B. quintana* infections.

The optimal treatment regimen for HIV-negative patients with suspected or confirmed *B. quintana* infection has not been established. Minimal published data exist regarding antimicrobial therapy for this infection, and in vitro susceptibility testing has proven unreliable (25). Nonetheless, on the basis of limited data, we believe it is reasonable to treat immunocompetent patients who have uncomplicated *B. quintana* bacteremia with at least 14 days of oral therapy with erythromycin, azithromycin, doxycycline, or tetracycline. In the 1993 Seattle outbreak, most patients had a satisfactory response to treatment with a beta-lactam agent followed by either erythromycin or azithromycin for 14 days (7). Although the number of patients identified with *B. quintana* endocarditis is small, most of these patients have required cardiac valve replacement despite intravenous antimicrobial therapy (5, 8, 26). Therefore, we recommend that patients with *B. quintana* endocarditis receive a more prolonged course of at least 4 to 6 months of antimicrobial therapy and cardiac valve replacement if needed. Further study is needed to determine the role of bactericidal agents, such as third generation cephalosporins or quinolones, as monotherapy or in combination with a bacteriostatic agent for treating invasive *B. quintana* infections.

Many aspects of the acquisition and pathogenesis of *B. quintana* infections, and specifically *B. quintana* infections among the homeless, are not well defined. Changes in the organism itself that have led to increased virulence may in part account for its reemergence; however, microbiologic data that can support or refute this hypothesis are lacking (27). The absence of recently identified cases in Seattle and in other areas with laboratories that use culture techniques appropriate for isolating *Bartonella* species suggests an episodic pattern of disease, with few or no cases occurring during interepidemic periods. It seems clear, however, that this most recent emergence of an old disease is related, at least in part, to societal factors that have contributed to urban decay and the existence of large homeless populations in our cities. As with other emerging infectious diseases, further efforts to identify, evaluate, and control *B. quintana* infections among homeless persons are challenges that will require the coordinated effort of clinicians, microbiologists, and public health officials.

**Lisa A. Jackson, M.D., M.P.H.*, and David H. Spach, M.D.[†]**

*School of Public Health and Community Medicine, and the [†]Division of Infectious Diseases and the Department of Medicine, University of Washington, Seattle, Washington, USA

### References

1. Vinson JW, Varela G, Molina-Pasquel C. Trench fever. III. Induction of clinical disease in volunteers inoculated with *Rickettsia quintana* propagated on blood agar. Am J Trop Med Hyg 1969;18:713-22.
2. Slater LN, Welch DF. *Rochalimaea* species (recently renamed *Bartonella*). In: Mandell GL, Bennett JE, Dolin R, editors. Principles and practice of infectious diseases, 4th ed. New York: Churchill Livingstone, 1995:1741-7.
3. Welch DF, Pickett DA, Slater LN, Steigerwalt AG, Brenner DJ. *Rochalimaea henselae* sp. nov., a cause of septicemia, bacillary angiomatosis, and parenchymal bacillary peliosis. J Clin Microbiol 1992;30:275-80.
4. Koehler JE, Quinn FD, Berger TG, LeBoit PE, Tappero JW. Isolation of *Rochalimaea* species from cutaneous and osseous lesions of bacillary angiomatosis. N Engl J Med 1992;327:1625-31.
5. Spach DH, Callis KP, Paauw DS, et al. Endocarditis caused by *Rochalimaea quintana* in a patient infected with human immunodeficiency virus. J Clin Microbiol 1993;31:692-4.
6. Maurin M, Roux V, Stein A, Ferrier F, Viraben R, Raoult D. Isolation and characterization by immunofluorescence, sodium dodecyl sulfate-polyacrylamide gel electrophoresis, western blot, restriction fragment length polymorphism-PCR, 16S rRNA gene sequencing, and pulsed-field gel electrophoresis of *Rochalimaea quintana* from a patient with bacillary angiomatosis. J Clin Microbiol 1994;32:1166-71.
7. Spach DH, Kanter AS, Dougherty MJ, et al.*Bartonella* (*Rochalimaea*) *quintana* bacteremia in inner-city patients with chronic alcoholism. N Engl J Med 1995;332:424-8.
8. Drancourt M, Mainardi JL, Brouqui P, et al. *Bartonella* (*Rochalimaea) quintana* endocarditis in three homeless men. N Engl J Med 1995;332:419-23.
9. Stein A, Raoult D. Return of trench fever [letter]. Lancet 1995;345:450-1.
10. Jackson LA, Spach DH, Kippen DA, Sugg NK, Regnery RL, Sayers MH, Stamm WE. Seroprevalence to *Bartonella quintana* among patients at a community clinic in downtown Seattle. J Infect Dis 1996;173:1023-6.
11. Regnery RL, Olson JG, Perkins BA, Bibb W. Serologic response to "*Rochalimaea henselae*" antigen in suspected cat scratch disease. Lancet 1992;339:1443-5.
12. Waldvogel K, Regnery RL, Anderson BA, Caduff R, Caduff J, Nadal D. Disseminated cat-scratch disease: detection of *Rochalimaea henselae* in affected tissues. Eur J Pediatr 1994;153:23-7.
13. Dalton MJ, Robinson LE, Cooper J, Regnery RL, Olson JG, Childs JE. Use of *Bartonella* antigens for serologic diagnosis of cat-scratch disease at a national referral center. Arch Intern Med 1995;155:1670-6.
14. Elgart ML. Pediculosis. Dermat Clin 1990;8:219-28.
15. Tappero JW, Mohle-Boetani J, Koehler JE, et al. The epidemiology of bacillary angiomatosis and bacillary peliosis. JAMA 1993;269:770-5.
16. Zangwill KM, Hamilton DH, Perkins BA, et al. Cat scratch disease in Connecticut: epidemiology, risk factors, and evaluation of a new diagnostic test. N Engl J Med 1993;329:8-13.
17. Barnes PF, El-Hajj H, Preston-Martin S, et al. Transmission of tuberculosis among the urban homeless. JAMA 1996;275:305-7.
18. Nardell E. McInnis B, Thomas B, Weidhaus S. Exogenous reinfection with tuberculosis in a shelter for the homeless. N Engl J Med 1986;315:1570-5.
19. Filice GA, Englender SJ, Jacobson JA, et al. Group A meningococcal disease in skid rows: epidemiology and implications for control. Am J Public Health 1984;74:253-4.
20. Counts GW, Gregory DF, Spearman JG, et al. Group A meningococcal disease in the U.S. Pacific Northwest: epidemiology, clinical features, and effect of a vaccination control program. Rev Infect Dis 1984;6:640-8.
21. DeMaria A Jr, Browne K, Berk SL, et al. An outbreak of type I pneumococcal pneumonia in a men's shelter. JAMA 1980;244:1446-9.

22. Pedersen AHB, Spearman J, Tronca E, et al. Diphtheria on skid road, Seattle, Washington, 1972-75. Public Health Rep 1977;92:336-42.
23. Heath CW Jr, Zusman J. An outbreak of diphtheria among skid row men. N Engl J Med 1962;267:809-12.
24. Larson AM, Dougherty MJ, Nowowiejski DJ, Welch DF, Matar GM, Swaminathan B, Coyle MB. Detection of *Bartonella* (*Rochalimaea*) *quintana* by routine acridine orange staining of broth blood cultures. J Clin Microbiol 1994;32:1492-6.
25. Myers WF, Grossman DM, Wisseman CL. Antibiotic susceptibility patterns in *Rochalimaea quintana*, the agent of trench fever. Antimicrob Agents Chemother 1984;25:690-3.
26. Spach DH, Kanter AS, Daniels NA, et al. *Bartonella (Rochalimaea) quintana* species as a cause of culture-negative endocarditis. Clin Infect Dis 1995;20:1044-7.
27. Relman DA. Has trench fever returned? N Engl J Med 1995;332:463-4.

## References

[PDF_00298] Barnes P. F., el-Hajj H., Preston-Martin S., Cave M. D., Jones B. E., Otaya M., Pogoda J., Eisenach K. D. (1996). Transmission of tuberculosis among the urban homeless.. JAMA.

[PDF_00308] Counts G. W., Gregory D. F., Spearman J. G., Lee B. A., Filice G. A., Holmes K. K., Griffiss J. M. (1984). Group A meningococcal disease in the U.S. Pacific Northwest: epidemiology, clinical features, and effect of a vaccination control program.. Rev Infect Dis.

[PDF_00286] Dalton M. J., Robinson L. E., Cooper J., Regnery R. L., Olson J. G., Childs J. E. (1995). Use of Bartonella antigens for serologic diagnosis of cat-scratch disease at a national referral center.. Arch Intern Med.

[PDF_00312] DeMaria A., Browne K., Berk S. L., Sherwood E. J., McCabe W. R. (1980). An outbreak of type 1 pneumococcal pneumonia in a men's shelter.. JAMA.

[PDF_00269] Drancourt M., Mainardi J. L., Brouqui P., Vandenesch F., Carta A., Lehnert F., Etienne J., Goldstein F., Acar J., Raoult D. (1995). Bartonella (Rochalimaea) quintana endocarditis in three homeless men.. N Engl J Med.

[PDF_00290] Elgart M. L. (1990). Pediculosis.. Dermatol Clin.

[PDF_00304] Filice G. A., Englender S. J., Jacobson J. A., Jourden J. L., Burns D. A., Gregory D., Counts G. W., Griffiss J. M., Fraser D. W. (1984). Group A meningococcal disease in skid rows: epidemiology and implications for control.. Am J Public Health.

[PDF_00274] Jackson L. A., Spach D. H., Kippen D. A., Sugg N. K., Regnery R. L., Sayers M. H., Stamm W. E. (1996). Seroprevalence to Bartonella quintana among patients at a community clinic in downtown Seattle.. J Infect Dis.

[PDF_00249] Koehler J. E., Quinn F. D., Berger T. G., LeBoit P. E., Tappero J. W. (1992). Isolation of Rochalimaea species from cutaneous and osseous lesions of bacillary angiomatosis.. N Engl J Med.

[PDF_00322] Larson A. M., Dougherty M. J., Nowowiejski D. J., Welch D. F., Matar G. M., Swaminathan B., Coyle M. B. (1994). Detection of Bartonella (Rochalimaea) quintana by routine acridine orange staining of broth blood cultures.. J Clin Microbiol.

[PDF_00257] Maurin M., Roux V., Stein A., Ferrier F., Viraben R., Raoult D. (1994). Isolation and characterization by immunofluorescence, sodium dodecyl sulfate-polyacrylamide gel electrophoresis, western blot, restriction fragment length polymorphism-PCR, 16S rRNA gene sequencing, and pulsed-field gel electrophoresis of Rochalimaea quintana from a patient with bacillary angiomatosis.. J Clin Microbiol.

[PDF_00327] Myers W. F., Grossman D. M., Wisseman C. L. (1984). Antibiotic susceptibility patterns in Rochalimaea quintana, the agent of trench fever.. Antimicrob Agents Chemother.

[PDF_00301] Nardell E., McInnis B., Thomas B., Weidhaas S. (1986). Exogenous reinfection with tuberculosis in a shelter for the homeless.. N Engl J Med.

[PDF_00317] Pedersen A. H., Spearman J., Tronca E., Bader M., Harnisch J. (1977). Diphtheria on Skid Road, Seattle, Wash., 1972-75.. Public Health Rep.

[PDF_00279] Regnery R. L., Olson J. G., Perkins B. A., Bibb W. (1992). Serological response to "Rochalimaea henselae" antigen in suspected cat-scratch disease.. Lancet.

[PDF_00334] Relman D. A. (1995). Has trench fever returned?. N Engl J Med.

[PDF_00253] Spach D. H., Callis K. P., Paauw D. S., Houze Y. B., Schoenknecht F. D., Welch D. F., Rosen H., Brenner D. J. (1993). Endocarditis caused by Rochalimaea quintana in a patient infected with human immunodeficiency virus.. J Clin Microbiol.

[PDF_00331] Spach D. H., Kanter A. S., Daniels N. A., Nowowiejski D. J., Larson A. M., Schmidt R. A., Swaminathan B., Brenner D. J. (1995). Bartonella (Rochalimaea) species as a cause of apparent "culture-negative" endocarditis.. Clin Infect Dis.

[PDF_00265] Spach D. H., Kanter A. S., Dougherty M. J., Larson A. M., Coyle M. B., Brenner D. J., Swaminathan B., Matar G. M., Welch D. F., Root R. K. (1995). Bartonella (Rochalimaea) quintana bacteremia in inner-city patients with chronic alcoholism.. N Engl J Med.

[PDF_00272] Stein A., Raoult D. (1995). Return of trench fever.. Lancet.

[PDF_00291] Tappero J. W., Mohle-Boetani J., Koehler J. E., Swaminathan B., Berger T. G., LeBoit P. E., Smith L. L., Wenger J. D., Pinner R. W., Kemper C. A. (1993). The epidemiology of bacillary angiomatosis and bacillary peliosis.. JAMA.

[PDF_00236] Vinson J. W., Varela G., Molina-Pasquel C. (1969). Trench fever. 3. Induction of clinical disease in volunteers inoculated with Rickettsia quintana propagated on blood agar.. Am J Trop Med Hyg.

[PDF_00282] Waldvogel K., Regnery R. L., Anderson B. E., Caduff R., Caduff J., Nadal D. (1994). Disseminated cat-scratch disease: detection of Rochalimaea henselae in affected tissue.. Eur J Pediatr.

[PDF_00245] Welch D. F., Pickett D. A., Slater L. N., Steigerwalt A. G., Brenner D. J. (1992). Rochalimaea henselae sp. nov., a cause of septicemia, bacillary angiomatosis, and parenchymal bacillary peliosis.. J Clin Microbiol.

[PDF_00294] Zangwill K. M., Hamilton D. H., Perkins B. A., Regnery R. L., Plikaytis B. D., Hadler J. L., Cartter M. L., Wenger J. D. (1993). Cat scratch disease in Connecticut. Epidemiology, risk factors, and evaluation of a new diagnostic test.. N Engl J Med.

